# Meaning of Life and Well-Being: Preliminary Results of the Successful Aging Study

**DOI:** 10.1093/geroni/igaa057.369

**Published:** 2020-12-16

**Authors:** Zvi Gellis, Kim McClive-Reed, Bonnie Kenaley, Eunhae Kim

**Affiliations:** 1 University of Pennsylvania, Philadelphia, Pennsylvania, United States; 2 University of Pennsylvania, Menands, New York, United States; 3 Boise State University, Boise, Idaho, United States; 4 Texas State University, San Marcos, Texas, United States

## Abstract

Meaning in life for older persons has become a focal research point, with findings that a greater sense of meaning is associated with better outcomes on a range of health and well-being factors. Our study examined relationships between scores on several personality scales, including the Meaning in Life Questionnaire (Steger et al., 2009) and the WHO-5 Well-Being Index, a proxy measure of mood/depression. Community-dwelling members (N=535) of Osher Lifelong Learning Institutes aged 50 and up (mean age 71.4, SD = 6.93) at 3 U.S. sites completed surveys. Higher wellness levels were significantly correlated with increased resilience, optimism, life satisfaction, and presence of meaning in life, while lower levels were associated with greater searching for meaning in life. A multivariate linear regression model (F = 55.597, df = 4, p = .000, R = .566, R2 = .320) showed that wellness scores increased with higher scores in optimism (ß = .348, p =.000), resilience (ß = .183, p = .000), and presence of meaning in life (ß = .106, p = .019). However, searching for meaning in life significantly predicted decreases in wellness scores (ß = -.084, p=.019). These results support those of previous studies, suggesting that for older persons, an ongoing search for meaning in life is linked to negative outcomes than a perception of existing meaning in life. A variety of available interventions aimed at increasing meaning and purpose in life (Guerrero-Torelles et al., 2017) may contribute to better health and well-being in older adults.

